# 4524 Fighting Malaria, One Image at a Time: Using Computer Vision to Develop an Automated Vector Speciation Tool

**DOI:** 10.1017/cts.2020.159

**Published:** 2020-07-29

**Authors:** Sophia Diaz, Tristan Ford, Monet Slinowsky, Kiley Gersch, Ebenezer Armah, Karina Frank, Zachary Buono, Margaret Glancey, Adam Goodwin, Soumyadipta Acharya

**Affiliations:** 1 Johns Hopkins University; 2 VecTech, JHU-Whiting School of Engineering, CBID; 3 JHU-Whiting School of Engineering, CBID

## Abstract

OBJECTIVES/GOALS: Rapid and accurate identification of primary malaria vector species from collected specimens is the most critical aspect of effective vector surveillance and control. This interdisciplinary team of engineers aims to automate identification using a deep learning computer vision algorithm. METHODS/STUDY POPULATION: The team spent August of 2019 observing and participating in control and surveillance activities in Zambia and Uganda. They conducted >65 interviews with key stakeholders across 9 malaria control and surveillance sites, ranging from field and community health workers, to malaria researchers and Ministry of Health employees. Stakeholder feedback validated the need for a more accurate and efficient method of vector identification in order to more effectively deploy targeted malaria interventions. The team set forth in designing and prototyping a portable, automated field tool that could speciate mosquito vectors to the complex level using artificial intelligence. RESULTS/ANTICIPATED RESULTS: The team’s research demonstrated that accuracy, cost effectiveness, and ease of use would be critical to the successful adoption of the tool. Results of initial prototyping, usability studies, and stakeholder surveys were used to determine the tool’s minimal user specifications: 1) the ability to distinguish between *Anopheles Gambiae* and *Anopheles Funestus*, the two principal malaria vectors in the countries visited, 2) achieving an identification accuracy of ≥90% to the complex level, and 3) accessibility to the speciation data 3-7 days following vector collection. Next steps include optimizing the tool to deploy a minimal viable product for testing in Kenya by the summer of 2020. DISCUSSION/SIGNIFICANCE OF IMPACT: The accurate, high-quality surveillance enabled by this device would allow malaria control programs to scale surveillance to remote regions where an entomologist may not be available, allowing malaria programs to deploy effective interventions, monitor results, and prevent disease.

